# Proanthocyanidins Attenuation of Chronic Lead-Induced Liver Oxidative Damage in Kunming Mice via the Nrf2/ARE Pathway

**DOI:** 10.3390/nu8100656

**Published:** 2016-10-21

**Authors:** Miao Long, Yi Liu, Yu Cao, Nan Wang, Meng Dang, Jianbin He

**Affiliations:** 1 Key Laboratory of Zoonosis of Liaoning Province, College of Animal Science & Veterinary Medicine, Shenyang Agricultural University, Shenyang 110866, China; longjlau@126.com (M.L.); m15648566678@163.com (Y.C.); wn168168wn@163.com (N.W.); joydivisiondang@sina.com (M.D.); 2 College of Life Engineering, Shenyang Institute of Technology, Fushun 113122, China; 3 School of Chemical Engineering, Sichuan University of Science and Engineering, Zigong 643000, China; liuyi0961@sina.com

**Keywords:** proanthocyanidin, lead, oxidative damage, Nrf2/ARE pathway, liver, mice, apoptosis, ER stress

## Abstract

Lead is harmful for human health and animals. Proanthocyanidins (PCs), a natural antioxidant, possess a broad spectrum of pharmacological and medicinal properties. However, its protective effects against lead-induced liver damage have not been clarified. This study was aimed to evaluate the protective effect of PCs on the hepatotoxicity of male Kunming mice induced by chronic lead exposure. A total of 70 healthy male Kunming mice were averagely divided into four groups: control group, i.e., the group exposed to lead, the group treated with PCs, and the group co-treated with lead and PCs. The mice exposed to lead were given water containing 0.2% lead acetate. Mice treated in the PCs and PCs lead co-treated groups were given PC (100 mg/kg) in 0.9% saline by oral gavage. Lead exposure caused a significant elevation in the liver function parameters, lead level, lipid peroxidation, and inhibition of antioxidant enzyme activities. The induction of oxidative stress and histological alterations in the liver were minimized by co-treatment with PCs. Meanwhile, the number of Transferase-Mediated Deoxyuridine Triphosphate-Biotin Nick End Labeling (TUNEL)-positive cells was significantly reduced in the PCs/lead co-treated group compared to the lead group. In addition, the lead group showed an increase in the expression level of Bax, while the expression of Bcl-2 was decreased. Furthermore, the lead group showed an increase in the expression level of endoplasmic reticulum (ER) stress-related genes and protein (GRP78 and CHOP). Co-treated with PCs significantly reversed these expressions in the liver. PCs were, therefore, demonstrated to have protective, antioxidant, and anti-ER stress and anti-apoptotic activities in liver damage caused by chronic lead exposure in the Kunming mouse. This may be due to the ability of PCs to enhance the ability of liver tissue to protect against oxidative stress via the Nrf2/ARE signaling pathway, resulting in decreasing ER stress and apoptosis of liver tissue.

## 1. Introduction

Lead is a non-essential toxic heavy metal which has been a persistent public health problem in many countries around the world. Lead is an environmental toxicant which causes a broad range of adverse effects in both humans and animals [1]. In the human body, lead mainly accumulates in the nervous system, blood, digestive and cardiovascular systems, and also in the kidney and bone [2,3,4]. Lead has also been shown to inhibit growth [5,6]. Lead can cause oxidative damage in many tissues, including the brain, heart, liver, kidney, and reproductive organs [7,8]. Recent studies have shown that oxidative stress caused by lead may damage the molecular mechanism at the cellular level [9] by inducing reactive oxygen species (ROS), depleting antioxidant capacity and increasing levels of lipid peroxidation [10,11]. Since lead poisoning principally arises from lead-contaminated air, dust, and soils, and by the lead-based paints, fertilizers, automobiles, cosmetics, batteries, etc., people are easily exposed to lead. Therefore, it is difficult to determine whether lead poisoning in people is caused by excessive intake of lead unless clinical symptoms appear. Since it is known that lead has a non-physiological role in the body, so after a long period of exposure to lead, the best way reduce its damage to the cell is to exclude lead.

As lead is a multi-target toxicant, it exerts a toxic manifestation by oxidative free radicals that mediate the disruption of the delicate pro- and anti-oxidant balance existing in mammalian cells, and a therapeutic strategy to elevate the antioxidant defenses of the body may be of assistance in protecting from lead toxicity. Recently, several anti-oxidative approaches have been proposed to alleviate the symptoms of lead damage [12,13,14]. Herbal and natural products possess antioxidant properties, and antioxidant molecules of plant origin have been widely investigated as scavengers of free radicals and suppressors of lipid peroxide (LPO). In this regard, numerous studies have exhibited the antioxidant activities of several natural products against many toxic metals [15].

Proanthocyanidins (PCs), also named condensed tannins, are oligomers and polymers of flavan-3-ols, contain various amounts of catechin and epicatechin [16], widely distributed in the plant kingdom, appearing in fruits, vegetables, seeds, nuts, flowers, and bark [17], and especially extracted from grape seeds [18]. They are highly water soluble, easy to extract, rich in various plants, and can be absorbed naturally [19]. PCs are powerful natural antioxidants and are efficient free radical scavengers, whose anti-oxidative ability exceeds that of vitamins C and E [20]. PCs have been reported to possess a broad spectrum of pharmacological and medicinal properties against oxidative stress. Some studies have revealed that PCs exhibit a wide range of biological effects, including anti-inflammatory, anti-arthritic, and anti-allergic properties [21]. Moreover, some studies demonstrated that they also have the ability to alleviate oxidative stress and degenerative diseases caused by some toxicants and medicines [22,23]. Some studies demonstrated that PCs could relieve oxidative stress in the small intestinal mucosa and reduce the injury induced by indomethacin [24], reverse pentylenetetrazole-induced impaired performance in the Morris water maze, oxidative stress, mitochondrial ROS generation [25], and significantly attenuate the doxorubicin-induced mutagencity via suppression of oxidative stress [26]. Many studies also showed that PCs could protect the reproductive toxicity induced by arsenic [27], cadmium [28], and formaldehyde [29], and the liver damage induced by carbon tetrachloride [30], gibberellic acid [31], and zearalenone [22].

However, to our knowledge, there are no similar studies available on the protective effects of PC on lead-induced hepatotoxicity in mice. If PCs have the ability to protect the liver, a diet comprising PCs would serve as useful clinical medicine against lead toxicity. Therefore, this study was aimed to elucidate whether PCs can prevent oxidative stress-induced hepatotoxicity and its possible mechanism.

## 2. Experimental Section

### 2.1. Animals

Male Kunming mice (15 ± 0.5 g and three weeks old) were purchased from the China Medical University (Shenyang, China). Mice received food and water randomly. All stress factors were reduced to a minimum. The experiments have been allowed by the ethics committee for laboratory animal care (Animal Ethics Procedures and Guidelines of the People’s Republic of China) for the use of Shenyang Agricultural University, China (Permit No. SYXK<Liao>2011-0001).

### 2.2. Chemicals

Standard lead was purchased from the National Standard Material Research Center (Beijing, China). Lead acetate was purchased from Shanghai Biochemical Technology Co., Ltd. (Shanghai, China). PCs extracted from grape seeds (purity > 95%) were obtained from Zelang Medical Technology Company (Nanjing, China). The extract contained oligomeric proanthocyanidins (88.36%), catechin (6.68%), l-epicatechin (4.54%), and were diluted in distilled water before use. The mice anti-Nrf2 and anti-γ-GCS antibodies were acquired from Santa Cruz biotechnology (Dallas, TX, USA). The citatory number of the anti-Nrf2 antibody was sc-722, the anti-γ-GCS antibody was sc-22755, and the anti-GRP78 antibody was sc-1050. Anti-HO-1 and β-actin antibodies were obtained from Sangon Biotech (Shanghai, China); anti-CHOP antibody was purchased from Beyotime Biotech (Shanghai, China); these antibodies were all polyclonal antibodies. We also purchased the antibodies conjugated with the secondary goat anti-mouse and goat anti-rabbit horseradish peroxides (HRP) from Beijing Solarbio Science and Technology Co., Ltd. (Beijing, China). SYBR green RT-PCR kit from Takara (Otsu, Japan) and 4′,6-diamidino-2-phenylindole (DAPI) from Sigma Aldrich (St. Louis, MO, USA) were also employed. The primers for Nrf2, HO-1, γ-GCS, GRP78, CHOP, and β-actin were synthesized and purified by Sangon Biotech (Shanghai, China). Moreover, the preservation solution of RNA samples and the kits for total animal RNA extraction were obtained from Sangon Biotech Co., Ltd. (Shanghai, China). The kits for Revert Aid First Strand cDNA synthesis were purchased from MBI Fermentas (Burlington, ON, Canada); kits for the testing of glutathione peroxidase (GSH-Px), reduced glutathione (GSH), malondialdehyde (MDA), superoxide dismutase (SOD), alkaline phosphatase (ALP), alanine aminotransferase (ALT) and aspartate aminotransferase (AST) activities were obtained from Nanjing Jiancheng Bioengineering Institute (Nanjing, China).

### 2.3. Experimental Design and Treatment

Mice were allowed to acclimatize for one week prior to commencing experiments. Mice were given standard granulated food and drinking water and were divided randomly into four groups, as follows: control group (*n* = 7), mice were given 0.9% physiological saline at 1 mL/kg b.w.; lead acetate group (*n* = 21), mice were given 0.2% lead acetate in their drinking water for six weeks along with 0.9% saline at 1 mL/kg b.w; PC group (*n* = 21), mice were given PC in 0.9% physiological saline at 100 mg/kg b.w. for six weeks by oral gavage for six days every week and stopped for one day; lead co-treatment with PC group (*n* = 21), mice were given 0.2% lead acetate in drinking water with PC at a dose of 100 mg/kg b.w. every day for six weeks.

### 2.4. Determination of Lead in Serum and Liver Tissue of Mice

Whole blood (0.5 mL) and liver tissue (30 mg) samples were taken from mice and then mixed with 0.5 mol/L HNO_3_ to dissolve the residue. Lead content was determined by a graphite furnace atomic absorption spectrophotometer.

### 2.5. Determination of Serum Enzymes

From the serum samples, commercially available enzyme linked immunosorbent assay (ELISA) kits were used to test the activities of ALP, ALT, and AST. Experimental procedures were carried out according to the manufacturer’s instructions (Nanjing Jiancheng Institute of Biotechnology, Nanjing, China). In brief, the samples were transferred into a new 96-well plate containing substrates or buffer solution. After incubation at 37 °C, the plate was incubated for an additional time after adding a color developing agent and the absorbance at 510 or 520 nm was measured.

### 2.6. Determination of Oxidative Stress in Mice

The liver tissue was weighed and diluted to 10% liver homogenate and centrifuged for 15 min at 3000 rpm. The supernatant was removed and used for analysis. The contents of MDA and reduced GSH, and the activities of GSH-Px and SOD were determined according to the instructions of the manufacturer for the specific kit (Nanjing Jiancheng Institute of Biotechnology). The concentration of MDA was assayed by monitoring thiobarbituric acid reactive substance formation. According to the instructions of the reduced glutathione assay kit (Nanjing Jiancheng Institute of Biotechnology), the concentration of reduced GSH was detected by the Dinitrothiocyano benzene (DNTB) rate colorimetric method.

### 2.7. TUNEL Analysis of Apoptosis

Mice liver paraffin slices were prepared for TUNEL assay, which was performed by using a commercial kit in accordance with the manufacturer’s instructions. Briefly, the paraffin slices were fixed with dimethylbenzene for 15 min at the room temperature and washed with absolute ethyl alcohol twice, for three minutes each time, then washed with phosphate-buffered saline (PBS) for the third time, for five minutes. The fixed sections were incubated with 100 μL of 20 μg/mL proteinase K solution for 10 min. Subsequently, the 10 μL 5× reaction buffer, 38 μL ddH_2_O, 1 μL fluorescein isothiocyanate (FITC)-labelled dUTP, and 1 μL terminal deoxynucleotidyl transferase (TdT) enzyme solution was mixed and added to the surface of one slide and incubated at 37 °C, for 1 h, in the dark. The sample was then strained by DAPI for 8 min. The labelled slices were washed and photographed under a fluorescence microscope. The nucleus of any apoptotic cells were brown, and the other nuclei were blue.

### 2.8. Western Blot Assay

The total protein and nuclear protein in 100 mg of the liver tissue of mice were extracted using Radio-Immunoprecipitation Assay (RIPA) lysis solution and a nuclear/cytoplasm protein extraction kit (Beyotime Biotech, Shanghai, China). The Bicinchoninic acid (BCA) protein assay kit (Beyotime, Shanghai, China) was used to determine the protein concentrations, and equivalent amounts of proteins (30 μg/lane) were loaded onto 12% sodium dodecyl sulfate-polyacrylamide gel electrophoresis gels (SDS-PAGE). After the proteins were separated by SDS-PAGE, they were transferred to a Modified as polyvinylidene fluoride (PVDF) membrane. Antibodies then were added for rabbit anti-Nrf2 (1:1000), anti-HO-1 (1:1000), anti-γ-GCS (1:500), anti-Bcl-2 (1:1000), anti-Bax (1:1000), and β-actin (1:2000) polyclonal antibodies, and incubated overnight at 4 °C. On the second day, HRP-labeled goat anti rabbit IgG was added and incubated at room temperature for 2 h. An Electrochemical luminescence (ECL) chemical luminescence method was then used to detect the color reaction. Images were acquired and the results of the gray scale image were analyzed using a Bio-Rad gel imaging system. The relative expression levels of proteins in each group were represented by the ratio of the gray value of the target protein and the β-actin.

### 2.9. Gene Expression

The total RNA of the 100 mg of the liver tissue in each group was extracted using TRIzol reagent. Then, the purity and the quantity of the total RNA were measured via the quotient for optical density (OD) at 260/280 nm (1.8–2.0). The 4 μg of total RNA from each sample was treated with DNAse. After DNAse treatment, 3 μg of DNA-free RNA in a total volume of 20 mL was then reverse transcribed into cDNA using MBI Ferments Prime Script RT reagent kit (Burlington, ON, Canada) according to the manufacturer’s instructions. The cDNA was used as the template for further quantitative RT-PCR analysis. An ABI 7500 real-time PCR system and the SYBR Green PCR Kit were used to conduct real-time PCR. Each sample had been measured in triplicate. For qRT-PCR reactions, 2 μL product of cDNA, 0.4 μL reverse primers, 0.4 μL forward, 10 μL 2× SYBR^®^ Premix Ex TaqTM, 6.8 μL of RNase-free water, and 0.4 μL ROX Reference Dye II (50×). The conditions of conducting the PCR reaction included: at the initial stage, denaturing at 95 °C for 5 min, and then denaturing at 95 °C for 15 s, annealing at 56 °C for 30 s, and extension at 72 °C for 30 s. The amount of the template was measured based on the standard curve of quantitative analysis. The primers of Nrf2, γ-GCS, Bcl-2, Bax, GRP78, and CHOP were designed by using Primer 5 software (PREMIER Biosoft company, Palo Alto, CA, USA) (Table 1), and β-actin was employed as a house-keeping gene. The results were analyzed by using the 2^−ΔΔCT^ assay.

### 2.10. Statistical Analyses

All data were processed using SPSS 17 statistical software (IBM, Almon, NY, USA). The significance level of statistic differences between mean values was determined using one-way ANOVA, and a *p*-value < 0.05 was considered as significant.

## 3. Results

### 3.1. The Effect of PC on the Lead Content of Whole Blood and Liver Tissue in Mice

As shown in Table 2, the lead levels detected in the blood and liver tissue of the lead-treated animals were significantly higher compared with the control group (*p* < 0.05). The experimental model was successful as a system to determine the effects of lead in the presence and absence of PCs in mice. However, lead levels in the blood and liver tissue of mice co-treated with lead and PCs showed no significant difference compared with the animals treated only with lead (*p* > 0.05), indicating that PCs did not significantly improve the status of the lead in the blood and liver tissue of mice.

### 3.2. The Effect of PCs on the Serum Enzymes of Mice Exposed to Lead

As shown in Table 3, the activities of ALP, ALT, and AST in the serum of the lead-treated group were significantly increased compared to the control group (*p* < 0.05). However, the activity of three enzymes in the group co-treated with PCs was significantly lower compared with those of the lead-treated group. These activities of these enzymes were not significantly altered in the PCs-only treated group compared with the control group. Co-treatment with PCs significantly reduced the activities of ALP, ALT, and AST (*p* < 0.05). The ALP, ALT, and AST levels in the lead group were higher than the control group.

### 3.3. The Effect of PCs on Oxidative Stress-Related Factors in the Liver of Mice Exposed to Lead

As shown in Table 4, the activities of GSH-Px and SOD significantly increased in the co-treated lead and PC group compared with the group only treated with lead. The MDA levels in the group treated only with lead were also shown to be significantly increased (*p* < 0.05) and in comparison to PCs-co-treated animals. GSH was significantly increased (*p* < 0.05) in the group co-treated with lead and PCs compared with the animals only treated with lead. The levels of MDA were also significantly decreased (*p* < 0.05) in the co-treated group compared with the group treated only with lead. These results indicated that administration of PCs significantly improved the lipid peroxidation damage in the liver tissue in mice exposed to lead.

### 3.4. The Effect of PCs on the Liver Tissue Histopathological Variation of Mice Exposed to Lead

**Figure 1:**
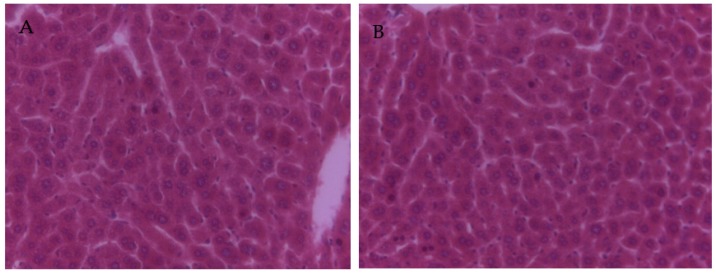

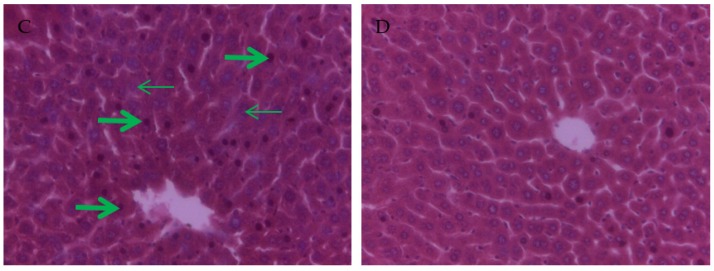
The effects of PCs on lead-induced liver histopathological changes in mice (original magnification of 400×). (**A**) Control group; (**B**) PCs group; (**C**) group administrated with lead; and (**D**) group co-treated with lead and PCs at a dose of 100 mg/kg. Thick arrow: nuclei pyknosis; Thin arrow: dissolved nuclei.

The results of the liver histopathological aspect are shown in Figure 1. Normal histological architecture was observed in the liver sections in the control group (Figure 1A) and PCs group (Figure 1B). However, in the lead group, it showed that the hepatic lobule structure was not integrated; the cells were arranged in a discontinuous and loose manner, the central vein tube wall was not integrated; the liver cells around the central vein were disordered; the liver cell size was not uniform, different sizes of vacuoles ere in the sytoplasmic; some nuclei of the hepatocytes were dissolved or pyknotic, and some hepatocytes were swollen and necrotic (Figure 1C). Animals co-treated with PCs largely improved the lead-induced histopathological changes in the liver tissue, showing clear borders of the hepatic tissue, a decrease of dissolved nuclei, and nuclei pyknosis (Figure 1D).

### 3.5. PCs Decreases Lead-Induced Apoptosis of Liver Cells

**Figure 2:**
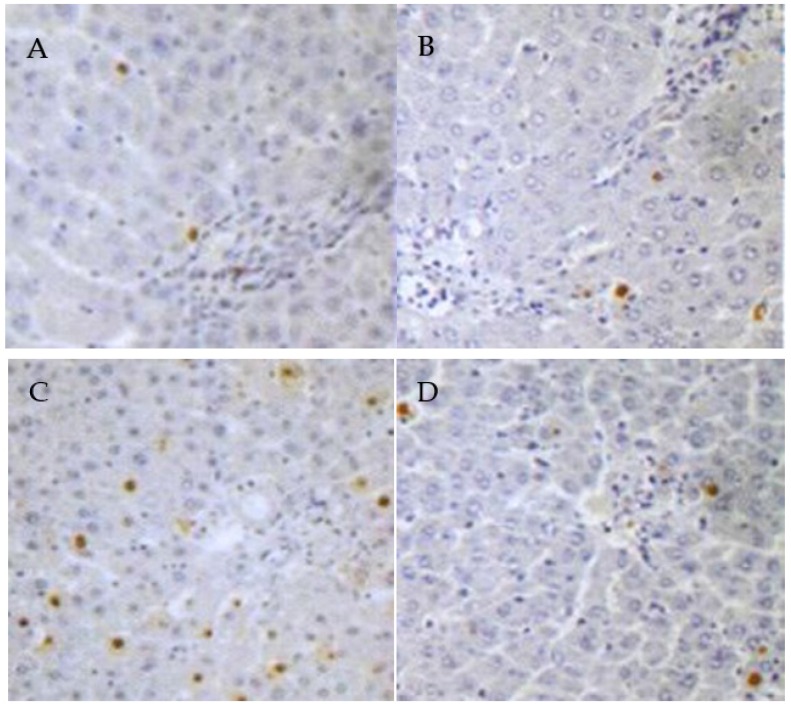
The effects of proanthocyanidins (PCs) on lead-induced liver apotosis. TUNEL-stained liver section (magnification, 200×), with brown granules indicating the positive cells. Mice were divided into the following groups: (**A**) control group; (**B**) PCs group; (**C**) group administrated with lead; (**D**) lead co-treated with PCs at a dose of 100 mg/kg.

Compared with the control group (Figure 2A), the TUNEL-positive cells were significantly increased in the lead group (*p* < 0.05) (Figure 2C), which was obviously attenuated by the co-administration of PCs (Figure 2D). However, the TUNEL-positive cells in the PCs group were lower than that in the lead group (*p* < 0.05).

### 3.6. The Effect of PC on the Expression of Bcl-2 and Bax in the Liver of Mice Exposed to Lead

**Figure 3:**
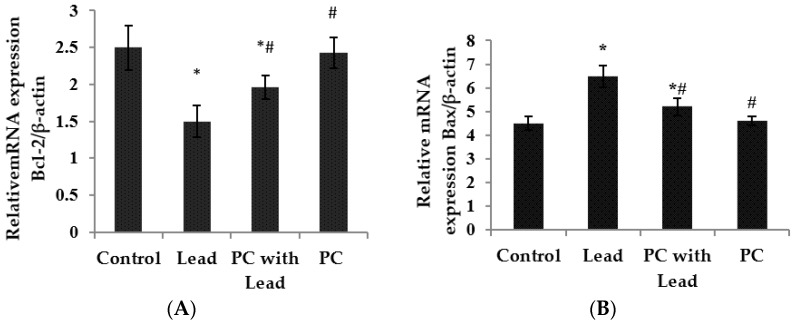
The effect of proanthocyanidin (PCs) on the mRNA levels of *Bcl-2* and *Bax* in the liver of mice exposed to lead. (**A**) *Bcl-2*; (**B**) *Bax*. All results are expressed as the mean ± SE (*n* = 7). * *p* < 0.05, significant change with respect to the control; ^#^ *p* < 0.05, significant change with respect to lead-treated mice.

**Figure 4:**
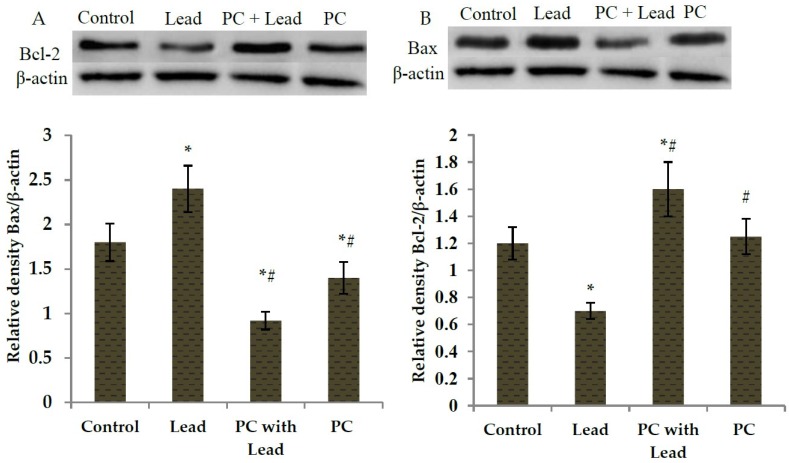
The effect of proanthocyanidins (PCs) on the protein expression levels of Bcl-2 and Bax in the liver of mice exposed to lead. (**A**) Bcl-2; (**B**) Bax. All results are expressed as the mean ± SE (*n* = 7). * *p* < 0.05, significant change with respect to the control; ^#^ *p* < 0.05, significant change with respect to lead-treated mice.

As we know, Bcl-2 has a role in cells that inhibits the signal which induces apoptosis, Bax has a role in promoting cell apoptosis, and in order to analyze whether PC protects against the toxicity caused by lead related to inhibiting cell apoptosis in the liver of mice, the expression of Bcl-2 and Bax associated with apoptosis was examined, As shown in Figure 3 and Figure 4, compared with the control group, the expression of Bcl-2 mRNA was down-regulated (Figure 3A) (*p* < 0.05), whereas the expression of Bax mRNA was up-regulated in the lead group (Figure 3B) (*p* < 0.05). However, compared with the lead group, the significant up-regulation of Bcl-2 mRNA (Figure 3A) (*p* < 0.05) and down-regulation of Bax (Figure 3B) (*p* < 0.05) were shown in the group co-treated with lead and PCs. Consistent with real-time PCR, the Western blot findings also showed low expression of Bcl-2 protein in lead-treated mice (Figure 4A) (*p* < 0.05), while moderate to strong expression was observed for Bax (Figure 4B) (*p* < 0.05). Compared with the lead-treated mice, those co-treated with PCs exhibited improvement, represented by increased expression of anti-apoptotic Bcl-2 protein and moderate expression of the pro-apoptotic protein Bax in the liver of mice (Figure 4A,B) (*p* < 0.05).

### 3.7. The Effect of PC on the Expression of Nuclear Nrf2, HO-1, and γ-GCS in the Liver of Mice Exposed to Lead

**Figure 5:**
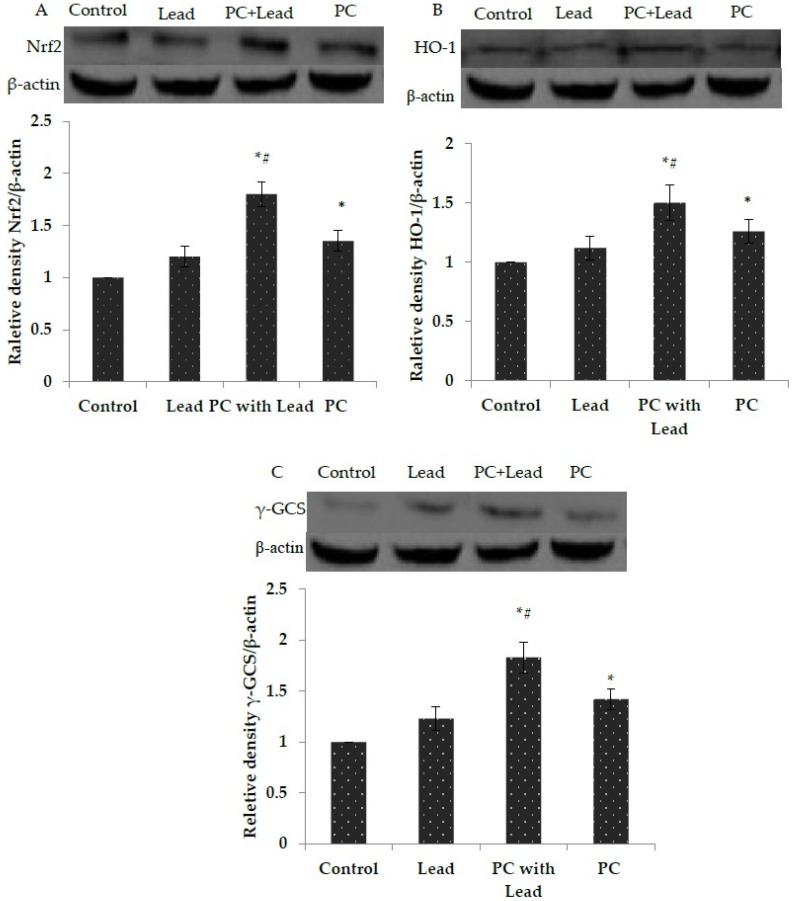
The effect of proanthocyanidins (PCs) on the protein expression levels of the nuclear Nrf2, HO-1, and γ-GCS protein in liver tissues of mice exposed to lead. (**A**) Nuclear erythroid 2-related factor 2 (Nrf2); (**B**) hemeoxygenase-1 (HO-1); and (**C**) γ-glutamyl cysteine synthetase (γ-GCS). All results are expressed as the mean ± SE (*n* = 7). * *p* < 0.05, significant change with respect to the control; ^#^ *p* < 0.05, significant change with respect to lead-treated mice.

**Figure 6:**
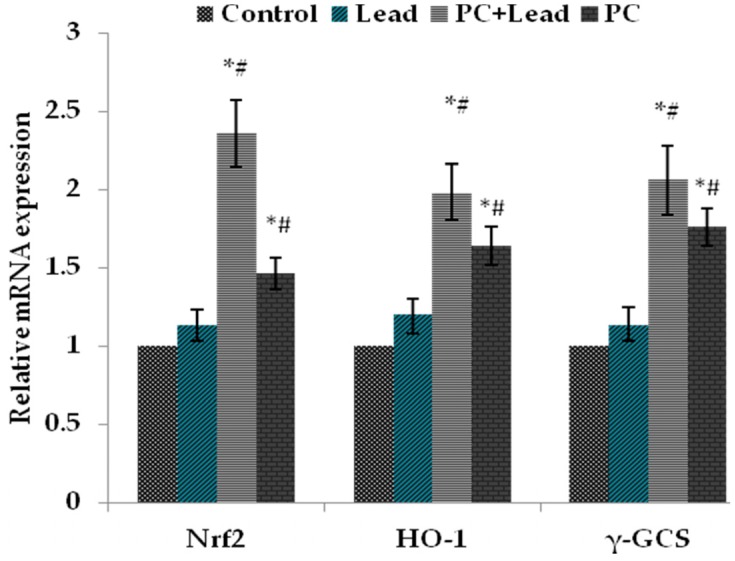
The effect of proanthocyanidins (PCs) on the mRNA expression levels of the nuclear *Nrf2*, *HO-1*, and *γ-GCS* in liver tissues of mice exposed to lead. All results are expressed as the mean ± SE (*n* = 7). * *p* < 0.05, significant change with respect to the control; ^#^ *p* < 0.05, significant change with respect to lead-treated mice.

To analyze whether Nrf2 activation plays a role in PC protection against the toxicity caused by lead, the expression of Nrf2 and Nrf2-target proteins, HO-1 and γ-GCS, in the liver of mice were measured. As shown in Figure 5, compared with the control group, the Western blot results showed that the nuclear Nrf2, HO-1, and γ-GCS protein expression levels in the lead-treated group were increased without significant difference (*p* > 0.05), while these protein expressions were increased with significant difference in the PCs group (*p* < 0.05). Compared with the lead group, all of their expressions in the lead/PCs co-treated group were increased with significant difference (*p* < 0.05) (Figure 5A–C). Meanwhile, as shown in Figure 6, the RT-PCR results showed that, compared with the control group, the nuclear *Nrf2*, *Ho-1*, and γ-GCS mRNA expression levels in the lead-treated group were increased without significant difference (*p* > 0.05), while these mRNA expressions were increased with significant difference in the PCs group. Compared with the lead group, the mRNA expressions of these genes in the lead/PCs co-treated group and in PCs group were increased with significant difference (*p* < 0.05).

### 3.8. The Effect of PC on the Expression of GRP78 and CHOP in the Liver of Mice Exposed to Lead

**Figure 7:**
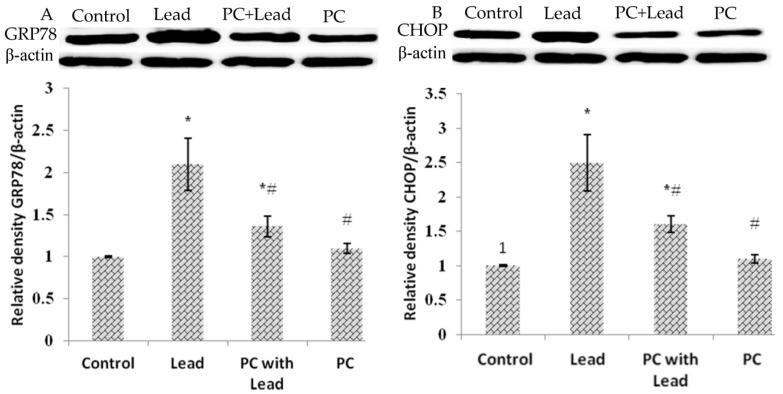
The effect of proanthocyanidins (PCs) on the protein expression levels of GRP78 and CHOP protein in liver tissues of mice exposed to lead. (**A**) GRP78; and (**B**) CHOP. All results are expressed as the mean ± SE (*n* = 7). * *p* < 0.05, significant change with respect to the control; ^#^ *p* < 0.05, significant change with respect to lead-treated mice.

**Figure 8:**
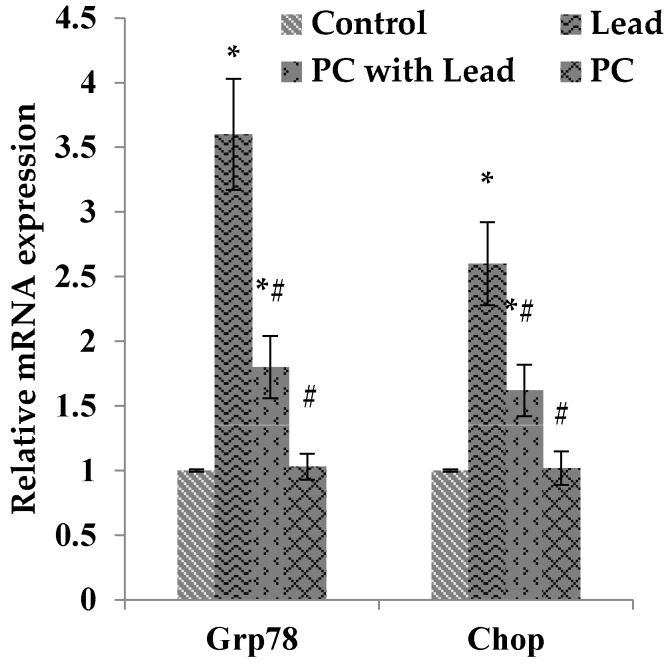
The effect of proanthocyanidins (PCs) on the mRNA expression levels of *Grp78* and *Chop* in liver tissues of mice exposed to lead. All results are expressed as the mean ± SE (*n* = 7). * *p* < 0.05, significant change with respect to the control; ^#^ *p* < 0.05, significant change with respect to lead-treated mice.

To analyze whether the protective effect of PCs on liver damage was related to inhibiting cell ER stress caused by lead, we measured the expression of GRP78 and CHOP, which were the ER stress-typical related proteins. As shown in Figure 7, compared with the control group, the Western blot results showed that the GRP78 and CHOP protein expression levels in the lead-treated group were increased with significant difference (*p* > 0.05), while these protein expressions were not significantly different in the PCs group. Compared with the lead group, all of the protein expressions in the lead/PCs co-treated group and PCs group were decreased with significant difference (*p* < 0.05) (Figure 7A,B). Meanwhile, as shown in Figure 8, the RT-PCR results showed that, compared with the control group, the *Grp78* and *Chop* mRNA expression levels in the lead-treated group were increased with significant difference (*p* < 0.05), while these mRNA expressions were not significantly different in the PCs group. Compared with the lead group, the mRNA expression of these genes in lead/PCs co-treated group and in PCs group were decreased with significant difference (*p* < 0.05).

## 4. Discussion

In the present study, the choice of the doses of lead and lead exposure time was based on preliminary experiments by Wang et al. [32,33], which had successfully established the model of chronic lead poisoning in mice. PCs were given to mice at a dose of 100 mg/kg body weight since this was reported to be the most effective dose in previous studies [22,34,35].

The toxicity of the lead has been relatively well studied, particularly in the context of neuro- and nephrotoxicity. As the liver is the central organ in the processing and removal of harmful substances from the body, it is also highly susceptible to damage. In addition, because the liver, via the portal vein, is the first organ exposed to internally-absorbed Pb and is one of the major organs implicated in the storage, biotransformation, and elimination of Pb, the liver must be the target organ of lead and liver will be damaged by lead when lead is taken into the body. Moreover, the liver is the body’s largest detoxification organ, a lot of detoxification enzymes, such as cysteine proteases, will be affected by lead. Previous studies have shown that lead exposure can cause oxidative damage in the liver by inhibiting the activities of antioxidant enzymes, decreasing the concentration of non-enzymatic antioxidants and increasing the concentration of reactive oxygen species [36,37,38]. Our results showed that lead acetate added to the drinking water for six weeks for mice resulted in severe histopathological changes in the liver tissue. In addition, the observed hepatotoxicity caused by lead acetate was accompanied with elevated activities of key liver enzymes, including AST, ALT, and ALP found in serum [39]. Our results of detection of serum biochemical parameters and pathology indicated that lead could accumulate in the liver and cause liver damage, and the reason for damage might be because lead mainly induces reactive oxygen species to attack the target organ and reduces a series of antioxidant enzymes, such as SOD, catalase, and the levels of some antioxidants (GSH) [40,41]. GSH is one of the main antioxidants found in liver tissue and it is central to the detoxification of the heavy metals in the liver. In the current study, it was observed that in the lead-treated group, the activity of GSH-Px was inhibited. As GSH-Px regulated the synthesis of GSH, the level of GSH was also shown to consequently decrease and the content of MDA, which was a product of lipid peroxidation, was shown to increase significantly. These results indicate that exposure to lead induced oxidative stress in the liver tissue of mice, which is in close agreement to previous observations reported by Senapati et al. [3] and Newairy et al. [42].

PCs are highly efficient natural antioxidants, their antioxidant activity is 50 times higher than that of vitamin E and 20 times that of vitamin C, and their effects have been reported in a wide range of studies [12,27,43,44,45,46,47]. The results of the meta-analysis by Li et al. [47] indicated that PCs could effectively improve the activity of anti-oxidative enzymes and reduce lipid peroxidation products. Previous studies showed that antioxidant can be useful in preventing hepatocellular damage by inhibiting lipid peroxidation by free radicals generated by heavy metals. From our data, it was shown that the mice co-treated with PCs saw increased levels of reduced GSH and higher activity of GSH-Px, and decreased the level of MDA, a result that might indicate that PCs could directly reduce phospholipid hydroperoxides within the membrane and lipoproteins by removing the ROS’ ability to inhibit lipid peroxidation of liver cells induced by lead. It may also be that PCs could improve the activity of GSH-Px by activating the intracellular antioxidant signaling pathway. In addition, the reduced GSH was at, or near, control levels; the reason might be that PCs may function by increasing the steady state levels of reduced GSH and/or its rate of synthesis, thereby conferring enhanced protection against oxidative stress. Combined with our result that PCs alleviated the liver tissue histopathological variation of mice exposed to lead, we concluded that PCs, via their anti-oxidant ability, protected the liver damage caused by lead.

Currently, Nrf2 is the key molecule which mediates the response of the endogenous antioxidant system. Activation of Nrf2 can promote the expression of antioxidant genes and induce synthesis of phase II detoxifying enzymes [48]. Studies have shown that Nrf2 plays a crucial role in cellular resistance to oxidation and exogenous damage [49,50]. Recent studies have indicated that activating the Nrf2/ARE pathway has a hepato-protective effect [51,52].

In our study, we found that the gene transcription level of Nrf2 and the expression protein level of Nrf2 were both increased in the lead group. This result indicated that the expression of Nrf2 could be induced by lead under chronic lead poisoning conditions. At the same time, the results indicated that under the chronic lead poisoning, in order to cope with the oxidative stress induced by lead, the cells opened the Nrf2/ARE signal pathway to compensate for the oxidation; however, the cells were still in a state of oxidative stress. When the mice were co-treated with PCs, the nuclear Nrf2 mRNA and protein expression were also increased with a significant difference compared with that in the control group and lead group. This result indicated that PCs could activate the expression of nuclear Nrf2 protein, indicating that the effect of PCs in antagonizing lead-induced oxidative stress in liver tissue was related to the Nrf2/ARE signaling pathway.

Previous studies have suggested that a variety of cytokines and antioxidants could counteract the action of such chemicals causing up-regulation of HO-1 and γ-GCS expression [53,54,55,56,57]. γ-GCS regulated by the Nrf2/ARE signaling pathway is the rate-limiting enzyme for synthesis of GSH [54]. Heme oxygenase 1 (HO-1), a known target of Nrf2-regulated transcription and an antioxidative, cytoprotective protein in different types of cells, plays an important role in cytoprotection when oxidative stress and cellular injury occurs, and Nrf2 directly regulates its expression [55,56]. Our results showed that PCs could up-regulate the Nrf2 downstream gene HO-1 and γ-GCS expression. This indicated that PCs could improve the expression of γ-GCS, resulting in accelerating GSH synthesis in the liver, then enhancing the ability to resist the oxidative stress in liver tissue caused by lead. Combined with our results that the reduced GSH was at, or near, control levels in mice co-treated with PCs, we demonstrated that PCs could increase the amount of reduced GSH through the Nrf2/ARE signaling pathway. Our results showed that PCs could improve the expression of HO1, resulting in increasing the ability of cells to fight against oxidative stress and then maintaining the redox balance. The present results demonstrated that promotion of HO-1 expression is required for PCs’ anti-oxidative effects. These results further illustrated that PCs played important roles as antioxidants and in detoxification by improving the expression of the antioxidant enzymes via the Nrf2/ARE signaling pathway. However, the molecular signaling pathway responsible for the expression of Nrf2 was not determined and is worthy of further investigation.

Apoptosis plays a key role in some metal-induced toxicity. Lead exposure can induce apoptosis of nerve cells [57], liver cells [58], and kidney cells [15] through inducing the depolarization and swelling of mitochondria, which leads to the release of cytochrome C, resulting in selective apoptosis [59]. As we know, Bcl-2 and Bax in apoptosis behave opposite to each other: Bax is a pro-apoptotic gene, while Bcl-2 is anti-apoptotic gene. Our results showed that the levels of Bcl-2 mRNA and protein were significantly down-regulated, while the expression levels of Bax were significantly up-regulated in the liver caused by lead. Our results demonstrated lead-induced liver cell apoptosis. Our results were consistent with previous investigations that hepatic apoptosis induced by low-dose exposure was associated with mitochondrial injury and changes in levels of apoptogenic proteins, such as Bcl-2, Bax, and caspase-3 [60,61]. However, PCs reversed these gene expressions, as our results showed that PCs up-regulated the anti-apoptotic gene Bcl-2 expression and down-regulated the pro-apoptotic gene Bax expression. Combined with the TUNEL assay results, we confirmed that PCs could effectively inhibit cell apoptosis in the liver.

Previous studies showed that lead could induce endoplasmic reticulum (ER) stress responses in the nervous system [62,63]. Our results demonstrated that lead induced the ER stress in the liver by increasing the expression of ER stress-related protein GRP78 and CHOP, results which were consistent with the previous study by Liu et al. [64]. Meanwhile, our results showed that mice co-treated with PCs decreased the expression of the two proteins, indicating that ER stress may be relieved by PCs treatment, which meant that PCs might protect the liver from damage by inhibiting ER stress in the liver.

Recent studies have provided evidence that PCs can be used in the treatment and prevention of diseases, such as atherosclerosis, gastric ulcers, cataracts, and diabetes. For the first time, our results suggested that the protective effect of PCs on lead-induced liver damage by inhibiting the ER stress and apoptosis is due, at least in part, to their anti-oxidant stress activity and their ability to modulate the Nrf2/ARE signaling pathway. These findings may be attributed to the manifold effects of PCs as functional foods in future applications.

## 5. Conclusions

In conclusion, PCs could protect against damage resulting from ER stress and apoptosis induced by chronic lead exposure in the liver tissue of mice. The hepatoprotective effect of PCs might be that PCs activated the expression of HO-1and γ-GCS via the Nrf2/ARE pathway, resulting in decreasing ER stress and apoptosis of the liver tissue cells.

## Figures and Tables

**Table 1:** Primers for real-time PCR analyses.

Gene	Accession No.	Primer Sequences (5′-3′)	Product Size/bp
Nrf2	NM_010902.3	F: TCCTATGCGTGAATCCCAAT	103 bp
		R: GCGGCTTGAATGTTTGTCTT	
Bcl-2	NM_009741.3	F: GTGGATGACTGAGTACCTGAACC	120 bp
		R: AGCCAGGAGAAATCAAACAGAG	
Bax	NM_007527.3	F: CGACCCGTCCTTTGAATTTCT	197 bp
		R: GCAAAGTAGAAGAGGGCAACCAC	
HO-1	NM_010442.2	F: GGGCTGTGAACTCTGTCCAAT	162 bp
		R: GGTGAGGGAACTGTGTCAGG	
γ-GCS	U85414.1	F: TGGATGATGCCAACGAGTC	185 bp
		R: CCTAGTGAGCAGTACCACGAATA	
GRP78	NC_000068.7	R: CACGTCCAACCCGAACGA	182 bp
		F: ATTCCAAGTGCGTCCGATG	
CHOP	NM007837.3	F: CAGCGACAGAGCCAGAATAA	84 bp
		R: TCAGGTGTGGTGGTGTATGAA	
β-actin	NM_007393.5	F: GTGCTATGTTGCTCTAGACTTCG	174 bp
		R: ATGCCACAGGATTCCATACC	

**Table 2:** Lead contents in serum and liver of mice in each group (*n* = 7, $\bar{X}$ ± s).

Group	Blood Lead Level/(μg/L)	Liver Lead Level/(μg/L)
Control	36.42 ± 17.48	0.88 ± 0.21
PCs	35.26 ± 13.36	0.82 ± 0.18
Lead	214.64 ± 36.24 *	13.44 ± 2.84 *
Lead with PCs	206.49 ± 34.92 *	11.21 ± 2.30 *

Note: PCs: Proanthocyanidins. * *p* < 0.05 vs. control group.

**Table 3:** The effect of PCs on serum enzyme levels in mice (*n* = 7, $\bar{X}$ ± s).

Group	ALP/(U/L)	ALT/(U/L)	AST/(U/L)
Control	129.47 ± 4.18	25.47 ± 7.16	57.92 ± 10.46
PC	124.42 ± 5.17 ^b,c^	23.42 ± 6.45 ^b,c^	50.21 ± 18.65 ^b,c^
Lead	246.48 ± 6.15 ^a,c^	59.23 ± 9.84 ^a,c^	86.29 ± 14.53 ^a,c^
Lead with PC	180.21 ± 7.96 ^a,b^	35.21 ± 6.80 ^a,b^	63.43 ± 11.24 ^a,b^

Note: With each row, means superscripted with different letters are significantly different (*p* < 0.05), ^a^ significantly different from the control *p* < 0.05; ^b^ significantly different from the lead group *p* <0.05; ^c^ significantly different from the lead co-treated with PCs group *p* < 0.05.

**Table 4:** The effect of PC on MDA, GSH content, and the activities of GSH-Px and SOD in the liver tissue of mice exposed to lead (*n* = 7, $\bar{X}$ ± s).

Group	MDA/(μmol/g Prot)	GSH/(mg/g Prot)	GSH-Px/(U/mg Prot)	SOD/(U/mg Prot)
Control	29.56 ± 4.78	18.47 ± 3.96	30.32 ± 3.69	132.63 ± 8.23
PC	23.44 ± 5.40	20.76 ± 3.45	36.42 ± 5.97	138.56 ± 6.24
Lead	64.32 ± 8.45 *	8.23 ± 2.14 *	11.73 ± 2.95 *	90.46 ± 4.23 *
Lead with PC	34.12 ± 5.36 ^#^	16.27 ± 4.10 ^#^	22.41 ± 2.74 *^,#^	116.45 ± 5.96 *^,#^

Note: Prot: protein. * *p* < 0.05 vs. the control group; ^#^ *p* < 0.05 vs. the lead group.

## References

[1] Tian F., Zhai Q., Zhao J., Liu X., Wang G., Zhang H., Zhang H., Chen W. (2012). *Lactobacillus plantarum* CCFM8661 alleviates lead toxicity in mice. Biol. Trace Elem. Res..

[2] Solon O., Riddell T.J., Quimbo S.A., Butrick E., Aylward G.P., Lou Bacate M., Peabody J.W. (2008). Associations between cognitive function, blood lead concentration, and nutrition among children in the central Philippines. J. Pediatr..

[3] Senapati S.K., Dey S., Dwivedi S.K., Swarup D. (2001). Effect of garlic (*Allium sativum* L.) extract on tissue lead level in rats. J. Ethnopharmacol..

[4] Chang W., Chen J., Wei Q.Y., Chen X.M. (2006). Effects of Brn-3a protein and RNA expression in rat brain following low level lead exposure during development on spatial learning and memory. Toxicol. Lett..

[5] Hamilton J.D., O’Flaherty J.E. (1995). Influence of lead on mineralization during bone growth. Fundam. Appl. Toxicol..

[6] Maboeta M.S., Reinecke A.J., Reinecke S.A. (1999). Effects of low levels of lead on growth and reproduction of the Asian earthworm *Perionyx excavatus* (Oligochaeta). Ecotoxicol. Environ. Saf..

[7] Ding Y., Gonick H.C., Vaziri N.D., Liang K., Wei L. (2001). Lead-induced hypertension III: Increased hydroxyl radical production. Am. J. Hypertens..

[8] Patra R.C., Swarup D., Dwidedi S.K. (2001). Antioxidant effects of α-tocopherol, ascorbic acid and l-methionine on lead-induced oxidative stress of the liver, kidney and brain in rats. Toxicology.

[9] Adonaylo V.N., Oteiza P.I. (1999). Pb^2+^ promotes lipid peroxidation and alteration in membrane physical properties. Toxicology.

[10] Farmand F., Ehdaie A., Roberts C.K., Sindhu R.K. (2005). Lead induced dysregulation of superoxide dismutases, catalase, glutathione peroxidase, and guanylate cyclase. Environ. Res..

[11] Gurer H., Ercal N. (2000). Can antioxidants be beneficial in the treatment of lead poisoning?. Free Radic. Biol. Med..

[12] Haleagrahara N., Jackie T., Chakravarthi S., Rao M., Kulur A. (2010). Protective effect of *Etlingera elatior* (torch ginger) extract on lead acetate—Induced hepatotoxicity in rats. J. Toxicol. Sci..

[13] Khalaf A.A., Moselhy W.A., Abdel-Hamed M.I. (2012). The protective effect of green tea extract on lead induced oxidative and DNA damage on rat brain. Neurotoxicology.

[14] Mabrouk A., Bel Hadj Salah I., Chaieb W., Ben Cheikh H. (2016). Protective effect of thymoquinone against lead-induced hepatic toxicity in rats. Environ. Sci. Pollut. Res. Int..

[15] Dkhil M.A., Al-Khalifa M.S., Al-Quraishy S., Zrieq R., Abdel Moneim A.E. (2016). *Indigofera oblongifolia* mitigates lead-acetate-induced kidney damage and apoptosis in a rat model. Drug Des. Dev. Ther..

[16] Malisch C.S., Lüscher A., Baert N., Engström M.T., Studer B., Fryganas C., Suter D., Mueller-Harvey I., Salminen J.P. (2015). Large variability of proanthocyanidin content and composition in Sainfoin (*Onobrychis viciifolia*). J. Agric. Food Chem..

[17] Mouradov A., Spangenberg G. (2014). Flavonoids: A metabolic network mediating plants adaptation to their real estate. Front. Plant Sci..

[18] Asl M.N., Hosseinzadeh H. (2009). Review of the pharmacological effects of *Vitis vinifera* (grape) and its bioactive compounds. Phytother. Res..

[19] Aruoma O.I., Sun B., Fujii H., Neergheen V.S., Bahorun T., Kang K.S., Sung M.K. (2006). Low molecular proanthocyanidin dietary biofactor Oligonol: Its modulation of oxidative stress, bioefficacy, neuroprotection, food application and chemoprevention potentials. Biofactors.

[20] Bagchi D., Garg A., Krohn R.L., Bagchi M., Tran M.X., Stohs S.J. (1997). Oxygen free radical scavenging abilities of vitamins C and E, and a grape seed proanthocyanidin extract in vitro. Res. Commun. Mol. Pathol. Pharmacol..

[21] Ariga T. (2004). The antioxidative function, preventive action on disease and utilization of proanthocyanidins. Biofactors.

[22] Long M., Yang S.H., Han J.X., Li P., Zhang Y., Dong S., Chen X., Guo J.Y., Wang J., He J.B. (2016). The protective effect of grape-seed proanthocyanidin extract on oxidative damage induced by zearalenone in Kunming mice liver. Int. J. Mol. Sci..

[23] Mansouri E., Panahi M., Ghaffari M.A., Ghorbani A. (2011). Effects of grape seed proanthocyanidin extract on oxidative stress induced by diabetes in rat kidney. Iran. Biomed. J..

[24] Cheung D.Y., Kim J.I., Park S.H., Kim J.K. (2014). Proanthocyanidin from grape seed extracts protects indomethacin-induced small intestinal mucosal injury. Gastroenterol. Res. Pract..

[25] Zhen J., Qu Z., Fang H., Fu L., Wu Y., Wang H., Zang H., Wang W. (2014). Effects of grape seed proanthocyanidin extract on pentylenetetrazole-induced kindling and associated cognitive impairment in rats. Int. J. Mol. Med..

[26] Attia S.M., Al-Bakheet S.A., Al-Rasheed N.M. (2010). Proanthocyanidins produce significant attenuation of doxorubicin-induced mutagenicity via suppression of oxidative stress. Oxid. Med. Cell. Longev..

[27] Li S.G., Ding Y.S., Niu Q., Xu S.Z., Pang L.J., Ma R.L., Jing M.X., Feng G.L., Liu J.M., Guo S.X. (2015). Grape seed proanthocyanidin extract alleviates arsenic-induced oxidative reproductive toxicity in male mice. Biomed. Environ. Sci..

[28] Hou F., Xiao M., Li J., Cook D.W., Zeng W., Zhang C., Mi Y. (2016). Ameliorative effect of grape seed proanthocyanidin extract on cadmium-induced meiosis inhibition during oogenesis in chicken embryos. Anat. Rec..

[29] Uluçam E., Bakar E. (2016). The effect of proanthocyanidin on formaldehyde-induced toxicity in rat testes. Turk. J. Med. Sci..

[30] Dai N., Zou Y., Zhu L., Wang H.F., Dai M.G. (2014). Antioxidant properties of proanthocyanidins attenuate carbon tetrachloride (CCl_4_)-induced steatosis/statuses and liver injury in rats via CYP2E1 regulation. J. Med. Food.

[31] Hassan H.A., Al-Rawi M.M. (2013). Grape seeds proanthocyanidin extract as a hepatic-reno-protective agent against gibberellic acid induced oxidative stress and cellular alterations. Cytotechnology.

[32] Wang C., Liang J., Zhang C., Bi Y., Shi X., Shi Q. (2007). Effect of ascorbic acid and thiamine supplementation at different concentrations on lead toxicity in liver. Ann. Occup. Hyg..

[33] Wang C., Zhang Y., Liang J., Shan G., Wang Y., Shi Q. (2006). Impacts of ascorbic acid and thiamine supplementation at different concentrations on lead toxicity in testis. Clin. Chim. Acta.

[34] Bagchi D., Garg A., Krohn R., Bagchi M., Bagchi D.J., Balmoori J., Stohs S.J. (1998). Protective effects of grape seed proanthocyanidins and selected antioxidants against TPA-induced hepatic and brain lipid peroxidation and DNA fragmentation, and peritoneal macrophage activation in mice. Gen. Pharmacol..

[35] Sato M., Maulik G., Ray P.S., Bagchi D., Das D.K. (1999). Cardio-protective effects of grape seed proanthocyanidin against ischemic reperfusion injury. J. Mol. Cell. Cardiol..

[36] Sandhir R., Gill K.D. (1995). Effect of lead on lipid peroxidation in liver of rats. Biol. Trace Elem. Res..

[37] Sharma S., Raghuvanshi S., Jaswal A., Shrivastava S., Shukla S. (2015). Lead acetate-induced hepatoxicity in Wistar rats: Possible protective role of combination therapy. J. Environ. Pathol. Toxicol. Oncol..

[38] Hasanein P., Kazemian-Mahtaj A., Khodadadi I. (2016). Bioactive peptide carnosin protects against lead acetate-induced hepatotoxicity by abrogation of oxidative stress in rats. Pharm. Biol..

[39] Sharma A., Sharma V., Kansal L. (2010). Amelioration of lead-induced hepatotoxicity by *Allium sativum* extracts in Swiss albino mice. Libyan J. Med..

[40] Chiba M., Shinohara A., Matsushita K., Watanabe H., Inaba Y. (1996). Indices of lead-exposure in blood and urine of lead-exposed workers and concentrations of major and trace elements and activities of SOD, GSH-Px and catalase/in their blood. Tohoku J. Exp. Med..

[41] Dafre A.L., Medeiros I.D., Müller I.C., Ventura E.C., Bainy A.C. (2004). Antioxidant enzymes and thiol/disulfide status in the digestive gland of the brown mussel *Perna perna* exposed to lead and paraquat. Chem. Biol. Interact..

[42] Newairy A.S., Abdou H.M. (2009). Protective role of flax lignans against lead acetate induced oxidative damage and hyperlipidemia in rats. Food Chem. Toxicol..

[43] Mansouri E., Khorsandi L., Abedi H.A. (2014). Antioxidant effects of proanthocyanidin from grape seed on hepatic tissue injury in diabetic rats. Iran. J. Basic Med. Sci..

[44] Bártíková H., Boušová I., Jedličková P., Lněničková K., Skálová L., Szotáková B. (2014). Effect of standardized cranberry extract on the activity and expression of selected biotransformation enzymes in rat liver and intestine. Molecules.

[45] Fu C., Wang H., Ng W.L., Song L., Huang D. (2013). Antioxidant activity and proanthocyanidin profile of *Selliguea feei* rhizomes. Molecules.

[46] Kresty L.A., Howell A.B., Baird M. (2011). Cranberry proanthocyanidins mediate growth arrest of lung cancer cells through modulation of gene expression and rapid induction of apoptosis. Molecules.

[47] Li S., Xu M., Niu Q., Xu S., Ding Y., Yan Y., Guo S., Li F. (2015). Efficacy of procyanidins against in vivo cellular oxidative damage: A systematic review and meta-analysis. PLoS ONE.

[48] Zhang X.S., Ha S., Wang X.L., Shi Y.L., Duan S.S., Li Z.A. (2015). Tanshinone IIA protects dopaminergic neurons against 6-hydroxydopamine-induced neurotoxicity through miR-153/NF-E2-related factor 2/antioxidant response element signaling pathway. Neuroscience.

[49] Niu Q., Mu L., Li S., Xu S., Ma R., Guo S. (2016). Proanthocyanidin Protects Human Embryo Hepatocytes from Fluoride-induced oxidative stress by regulating iron metabolism. Biol. Trace Elem. Res..

[50] Ye F., Li X., Li L., Lyu L., Yuan J., Chen J. (2015). The role of Nrf2 in protection against Pb-induced oxidative stress and apoptosis in SH-SY5Y cells. Food Chem. Toxicol..

[51] Jeong G.S., Lee D.S., Li B., Byun E., Kwon D.Y., Park H., Kim Y.C. (2010). Protective effect of sauchinone by up-regulating heme-oxygenase-1 via the P38 MAPK and Nrf2/ARE pathways in HepG2 cells. Planta Med..

[52] Krajka-Kuźniak V., Paluszczak J., Oszmiański J., Baer-Dubowska W. (2014). Hawthorn (*Crataegus oxyacantha* L.) bark extract regulates antioxidant response element (ARE)-mediated enzyme expression via Nrf2 pathway activation in normal hepatocyte cell line. Phytother. Res..

[53] Borroz K.I., Buetler T.M., Eaton D.L. (1994). Modulation of gamma-glutamylcysteine synthetase large subunit mRNA expression by butylated hydroxyanisole. Toxicol. Appl. Pharmacol..

[54] Jin X., Liu Q., Jia L., Li M., Wang X. (2015). Pinocembrin attenuates 6-OHDA-induced neuronal cell death through Nrf2/ARE pathway in SH-SY5Y cells. Cell. Mol. Neurobiol..

[55] Shi M.M., Kugelman A., Iwamoto T., Tian L., Forman H.J. (1994). Quinine-induced oxidative stress elevates glutathione and induces gamma-glutamylcysteine synthetase activity in rat lung epithelial L2 cells. J. Biol. Chem..

[56] Wang Y., Fang J., Huang S., Chen L., Fan G., Wang C. (2013). The chronic effects of low lead level on the expressions of Nrf2 and Mrp1 of the testes in the rats. Environ. Toxicol. Pharmacol..

[57] Ye F., Li X., Li L., Yuan J., Chen J. (2016). t-BHQ provides protection against lead neurotoxicity via Nrf2/HO-1 pathway. Oxid. Med. Cell. Longev..

[58] Dewanjee S., Dua T.K., Khanra R., Das S., Barma S., Joardar S., Bhattacharjee N., Zia-Ul-Haq M., Jaafar H.Z. (2015). Water Spinach, *Ipomoea aquatic* (Convolvulaceae), Ameliorates lead toxicity by inhibiting oxidative stress and apoptosis. PLoS ONE.

[59] He L., Poblenz A.T., Medrano C.J., Fox D.A. (2000). Lead and calcium produce rod photoreceptor cell apoptosis by opening the mitochondrial permeability transition pore. J. Biol. Chem..

[60] Yuan G., Dai S., Yin Z., Lu H., Jia R., Xu J. (2014). Sub-chronic lead and cadmium co-induce apoptosis protein expression in liver and kidney of rats. Int. J. Clin. Exp. Pathol..

[61] Abdel Moneim A.E. (2016). *Indigofera oblongifolia* prevents lead acetate-induced hepatotoxicity, oxidative stress, fibrosis and apoptosis in rats. PLoS ONE.

[62] Qian Y., Tiffany-Castiglioni E. (2003). Lead-induced endoplasmic reticulum (ER) stress responses in the nervous system. Neurochem. Res..

[63] Zhang Y., Sun L.G., Ye L.P., Wang B., Li Y. (2008). Lead-induced stress response in endoplasmic reticulum of astrocytes in CNS. Toxicol. Mech. Methods.

[64] Liu C.M., Zheng G.H., Ming Q.L., Sun J.M., Cheng C. (2013). Protective effect of quercetin on lead-induced oxidative stress and endoplasmic reticulum stress in rat liver via the IRE1/JNK and PI3K/Akt pathway. Free Radic. Res..

